# Autonomic Flexibility and Early Treatment Success: Heart Rate Variability Predicts Remission in First-Episode Psychosis

**DOI:** 10.1093/schbul/sbaf191

**Published:** 2025-10-28

**Authors:** Judith Rohde, Samantha Weber, Mateo de Bardeci, Aygün Ertuğrul, Grammato Amexi, Eva Schultz, Sebastian Olbrich

**Affiliations:** Department of Adult Psychiatry and Psychotherapy, Psychiatric University Hospital Zurich, 8032 Zurich, Switzerland; Department of Adult Psychiatry and Psychotherapy, Psychiatric University Hospital Zurich, 8032 Zurich, Switzerland; Department of Adult Psychiatry and Psychotherapy, Psychiatric University Hospital Zurich, 8032 Zurich, Switzerland; Department of Adult Psychiatry and Psychotherapy, Psychiatric University Hospital Zurich, 8032 Zurich, Switzerland; Department of Psychiatry, Hacettepe University Faculty of Medicine, 06230 Ankara, Turkey; Department of Adult Psychiatry and Psychotherapy, Psychiatric University Hospital Zurich, 8032 Zurich, Switzerland; Department of Adult Psychiatry and Psychotherapy, Psychiatric University Hospital Zurich, 8032 Zurich, Switzerland; Department of Adult Psychiatry and Psychotherapy, Psychiatric University Hospital Zurich, 8032 Zurich, Switzerland

**Keywords:** heart rate variability, first-episode psychosis, remission prediction, autonomic nervous system, ECG biomarkers, physiological markers

## Abstract

**Background and Hypothesis:**

Heart-rate variability (HRV) is a low-cost marker of autonomic regulation and a potential prognostic biomarker in psychosis. We hypothesized that HRV markers recorded at admission are associated with remission at discharge in patients with suspected first-episode psychosis.

**Study Design:**

In this retrospective study, 78 inpatients (mean age: 30.8 years; 45% female) admitted to the Psychiatric University Hospital Zurich between 2018 and 2024 underwent a 2-min electrocardiogram on admission. Frequency-domain indices (log-transformed high-frequency [HF] and low-frequency [LF] power) and the linear slope of heart rate over time (beats per minute [BPM] slope) were extracted. Remission status at discharge was determined from clinician-rated scales and clinical evaluation. Binomial logistic regression with 5-fold stratified cross-validation assessed predictive accuracy (area under the receiver operating curve, AUC).

**Study Results:**

Twenty-six patients (33.3 %) remitted. Sex was not associated with outcome. In the initial model, log HF (*P* = .011) and BPM slope (*P* = .014) predicted remission, whereas log LF and mean BPM did not. The final model retaining log HF and BPM slope achieved an AUC of 0.714. Lower log HF and more rapidly declining BPM slope at admission were linked to higher odds of remission.

**Conclusions:**

An HRV profile combining low vagally mediated HF power with rapid heart rate decrease predicted short-term remission, supporting the autonomic-flexibility framework. HRV may aid early treatment stratification, but findings require prospective validation with larger samples, respiratory monitoring, and standardized interventions.

## Introduction

Psychotic disorders are serious mental health conditions, with an estimated global point prevalence of 3.89 per 1000 individuals and a lifetime prevalence of 7.49 per 1000 individuals, affecting millions worldwide.^1^ These disorders, often begin in early adulthood,^2^ are associated with high rates of psychiatric and somatic comorbidities,^3^ and carry an elevated risk of suicide.^4^ Among individuals receiving treatment following a first episode of psychosis, remission rates are 58% and recovery rates 38%.^5^ Despite this, there remains a significant treatment gap, with only 29% of affected individuals receiving appropriate care.^6^

Several factors have been identified as predictors of symptomatic remission, including demographic factors (eg, female sex, treatment onset, or symptom severity^7^), and neurophysiological biomarkers derived from neuroimaging studies^8^ or digital biomarkers.^9^ However, these approaches have shown limited predictive value^10^ and often require specialized equipment,^9^ limiting their utility for understanding of the biological mechanisms underlying relapse.^11^

Given the limitations of current biomarkers, an alternative approach to identifying predictive biomarkers could be the analysis of heart rate variability (HRV). HRV describes fluctuations in time between consecutive heartbeats and is widely recognized as a non-invasive marker of autonomic nervous system activity. It reflects the dynamic interplay between sympathetic and parasympathetic influences on heart function, with higher HRV indicating better adaptability and autonomic regulation, while lower HRV is associated with autonomic dysfunction and potential health impairments.^12^ HRV can be assessed using multiple analytical approaches, including time-domain, frequency-domain, and nonlinear methods, with electrocardiography (ECG) being the standard measurement tool.^12^ Frequency-domain analysis estimates the distribution of heart rate power across 4 bands—ultra-low, very-low (VLF), low (LF), and high frequency (HF)—as defined by international guidelines.^13^

Psychiatric disorders, including schizophrenia, have been associated with autonomic dysregulation and alterations in HRV,^14–17^ and some evidence links HRV to symptom severity^18^ or changes following interventions such as electroconvulsive therapy.^19^ Nonetheless, HRV’s role as a predictor of short-term clinical outcomes in first-episode psychosis remains underexplored. Together with the marked heterogeneity in illness trajectories and the large unmet clinical need, there is a pressing demand for low-cost, non-invasive tools to support personalized treatment planning and early prognostic decision-making.

To address this gap, the present study investigates whether frequency-domain HRV parameters at hospital admission are associated with remission status at discharge in individuals with suspected first-episode psychosis in a real-world setting. We hypothesize that HRV markers at admission will be associated with treatment outcomes, supporting their utility as physiological indicators for early prognosis in personalized psychosis care.

## Methods

### Study Design and Participants

This retrospective observational study included 78 patients with suspected first-episode psychosis (ICD-10: any F2 diagnosis) who were admitted to the Psychiatric University Hospital Zurich, Switzerland for routine inpatient treatment between 2018 and 2024. We analyzed patient data, including ECG, validated clinician-administered assessments, and discharge letters.

### Procedure

Upon admission, each patient underwent a simple bipolar ECG recording during resting state as part of routine diagnostic evaluations. For HRV analysis, 120 s of artifact-free ECG data were used. Details of the HRV processing pipeline are provided below.

Clinician-administered assessments included standardized and validated rating scales. These comprised the Clinical Global Impressions Scale—Severity (CGI-S), which evaluates the severity of mental illness on a 7-point scale (1 = normal/not ill to 7 = extremely ill),^20^ and the Health of the Nation Outcome Scales (HoNOS), a 12-item instrument assessing behavioral, cognitive, symptomatic, and social functioning problems on a scale from 0 (no problem) to 4 (severe problem), with a maximum total score of 48.^21^ Based on these clinical assessments and additional validated psychometric instruments, a final clinical judgment regarding remission status was documented in the discharge letter, which served as the outcome definition in this study.

### Data Analysis

#### ECG Data Analysis

HRV was computed using the DeepPsy Analysis Pipeline (version 1.1.7; *DeepPSY*, Zollikerberg, CH),^22^ which follows the Kubios HRV standard analysis framework (https://www.kubios.com/). ECG peak positions were visually inspected to ensure accuracy before processing. RR intervals (time between consecutive R peaks; ms) were derived from the verified peaks, interpolated using cubic splines, and detrended using a smoothness priors approach. Power spectral density was then estimated using Welch’s method. Standard HRV metrics were extracted, including VLF, LF, HF, LF/HF ratio, beats per minute (BPM), and BPM slope, calculated for both the full ECG trace and the first 120 s. Classical time-domain indices such as standard deviation of normal-to-normal intervals (SDNN) or root mean square of successive differences (RMSSD) are not available in this certified software.

### Sample Characteristics

Descriptive analyses were conducted to characterize the sample’s basic demographic and clinical characteristics and to examine gender differences across variables, as observed previously.^14^ Initially, percentages, means, and SE were calculated for the total sample using SPSS (IBM SPSS Statistics, Version 30.0.0.0). Subsequently, gender-specific descriptive statistics were obtained.

To determine appropriate group comparisons, the normality of each continuous variable was assessed using the Shapiro–Wilk test. Independent-samples *t*-tests were applied for normally distributed variables; Welch’s *t*-test was used when variances were unequal (Levene’s test). Non-normally distributed variables were compared using the Mann–Whitney U test, and group differences were reported using mean ranks. Associations between gender and remission status were analyzed using chi-square tests and cross-tabulations.

All 78 participants were included in the analyses with no missing data.

### Prediction of Remission

To examine whether remission status, determined from discharge reports based on standardized assessments and clinical judgment, could be predicted from ECG-derived parameters, a binomial logistic regression model was fitted. Several predictors exhibited skewed distributions and were therefore log-transformed (HF and LF) to improve linearity and model interpretability. Remission was initially coded as a 3-level variable; for analysis, partial and no remission were combined into a single category.

An initial model including 4 ECG-based predictors—log(HF), log(LF), BPM, and BPM slope—was evaluated using 5-fold stratified cross-validation. The ratio of LF and HF was not included to avoid interpretability problems due to the strong correlation with LF and HF. Based on model performance and conceptual relevance, log(HF) and BPM slope were retained for the final model, which was likewise trained using 5-fold cross-validation and optimized for the area under the ROC curve (AUC). Out-of-fold predictions were used to compute a cross-validated ROC curve. All preprocessing steps, including variable transformation, handling of missing data, and recoding of remission status, were completed prior to model fitting.

### Ethics Approval

Participants provided written informed consent for the use of their data. As the study did not fall under the Swiss Human Research Act, formal approval from the Cantonal Ethics Committee of Zurich was not required (BASEC No. Req-2020-00840). The study was not registered in a clinical trial registry, as retrospective observational studies are not subject to mandatory registration.

## Results

### Sample Characteristics

Our sample comprised 78 patients with a mean age of 30.79 years. Of these, 45% were female (*n* = 35), and 55% were male (*n* = 43). Table 1 presents detailed demographic, clinical, and ECG characteristics at admission. All patients received standard inpatient care, including regular consultations with a psychiatrist, at least 1 antipsychotic medication, and adjunctive therapies such as occupational therapy. At discharge, 33.3% of patients (*n* = 26) were rated as being in remission, while 66.7% (*n* = 52) showed partial or no remission. Among female patients, 28.6% (*n* = 10) achieved remission compared to 37.2% (*n* = 16) of male patients. No significant sex during hospitalization, all patients received atypical antipsychotic medication. In the partial/non-remission group (*n* = 52), 39 (75%) were discharged with a single antipsychotic, 8 (15%) with more than 1 antipsychotic, and 5 patients (10%) without an antipsychotic. The most frequently prescribed drug was olanzapine (23%), followed by risperidone (19%) and aripiprazole (15%). In the remission group (*n* = 26), 22 patients (85%) received 1 antipsychotic, 1 (4%) more than 1 antipsychotic, and 3 patients (12%) no antipsychotic. The most frequently prescribed drug was again olanzapine (27%), followed by risperidone (23%) and aripiprazole (15%). All antipsychotic prescriptions were documented as clinically adequate doses and durations at discharge. There was no significant difference between groups in the distribution of patients receiving no, one, or more than one antipsychotic medication (χ^2^ test, *P* > .05).

**Table 1:** Demographic and Clinical Variables

Scores at admission	Full sample (*n* = 78)		Female patients (*n* = 35)		Male patients (*n* = 43)	
	Mean	SE	Mean	SE	Mean	SE
Age (years)	30.79	1.194	32.86^*^	2.244	29.12^*^	1.128
CGI–S	5.34	0.112	5.20^*^	0.191	5.46^*^	0.126
HoNOS	20.16	1.074	19.97^*^	1.586	20.32^*^	1.478
BPM	78.58	1.684	78.51^*^	2.465	78.63^*^	2.330
BPM slope	−0.1058	0.16747	−0.0857^*^	0.23771	−0.1221^*^	0.23668
HF (ms^2^)	1064.354	220.402	1448.666^*^	449.975	751.542^*^	151.460
LF (ms^2^)	694.022	155.550	879.413^*^	324.223	543.123^*^	99.955
LF/HF	2.228	0.462	2.218^*^	0.768	2.236^*^	0.568

Abbreviations: CGI–S = Clinical Global Impressions Scale-Severity; HoNOS = Health of the Nation Outcome Scales. For non-normally distributed variables, mean ranks were used.

^^*^^No significant sex-based differences were observed (*P* > .05), based on independent samples *t*-tests and Mann–Whitney U tests, respectively.

Of the 78 patients included in the study, at discharge, 21 (27%) were diagnosed with schizophrenia (ICD-10: F20.x), 32 (41%) with acute and transient psychotic disorders (F23.x), and 3 (4%) with schizoaffective disorder (F25.x). The remaining 22 (28%) patients were diagnosed with other psychiatric disorders, such as affective or substance use disorders. Prediction of remission.

An initial model using binomial logistic regression included all 4 ECG parameters (log(HF), log(LF), BPM, and BPM slope). Table 2 presents the coefficients of the initial logistic regression model with all 4 ECG-based predictors. Among these, log(HF) and BPM slope were significantly associated with remission status (*P* < .05), while the other predictors did not reach statistical significance.

**Table 2:** Coefficients of the Initial Binomial Logistic Regression Model Predicting Remission Status from ECG-Derived Parameters

Coefficients	Estimate	SE	*Z*-value	*P*-value
Intercept	4.24683	3.15245	1.347	.1779
BPM	−0.03064	0.02564	−1.195	.2322
BPM slope	−0.50799	0.20721	−2.452	.0142
log (HF)	−0.70551	0.27859	−2.532	.0113
log (LF)	0.21940	0.24946	0.879	.3791

Model fit: residual deviance = 84.16 on 73 df; AIC = 94.16.

Based on preliminary model performance and conceptual relevance, log(HF) and BPM slope were retained for the final prediction model, which achieved an AUC of 0.714 (Figure 1). Table 3 shows the coefficients of the final binomial regression model. Figure 2 illustrates BPM slope and log(HF) for no/partial remission and remission.

**Figure 1:**
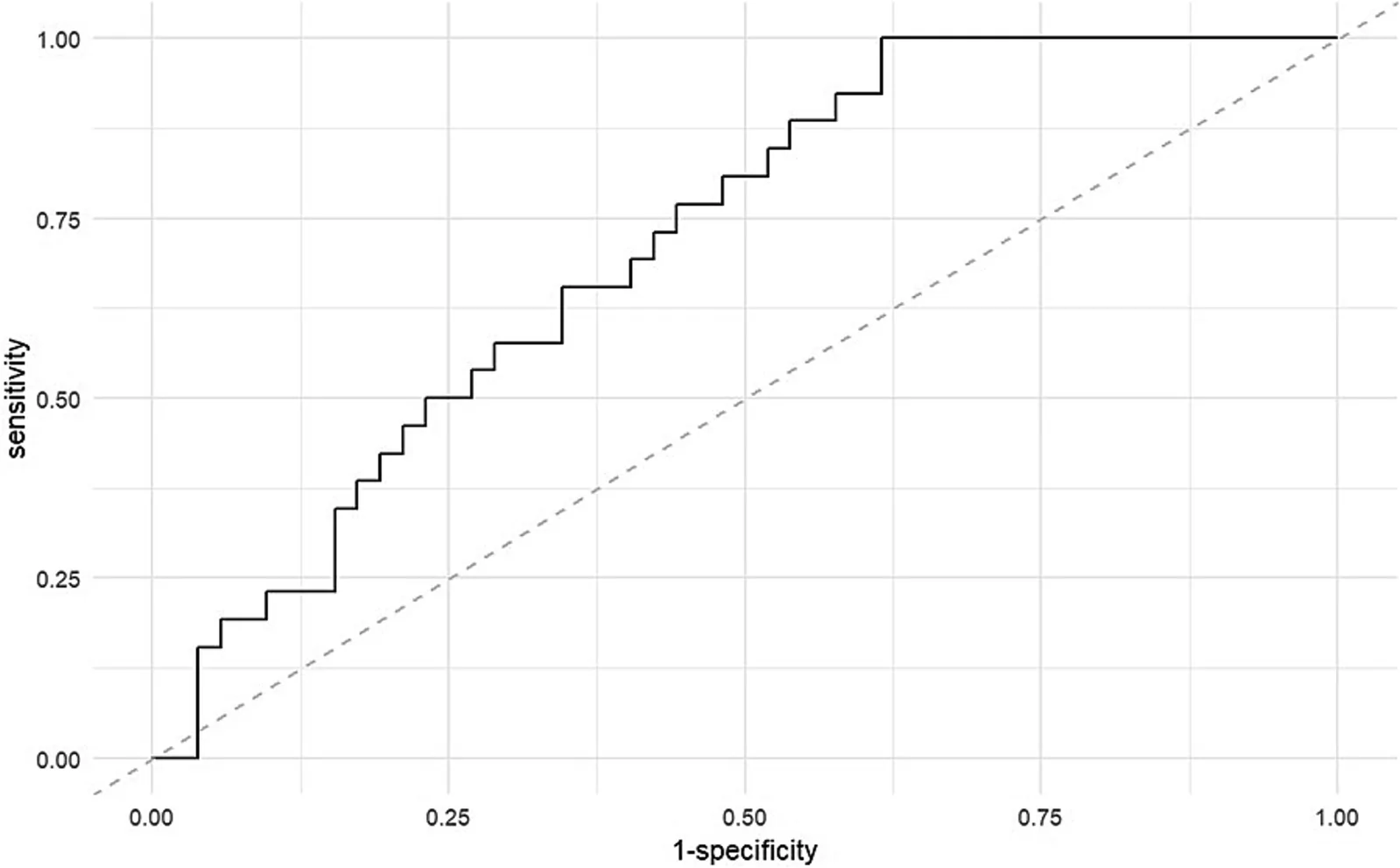
Cross-validated ROC curve for the final logistic regression model predicting remission status based on log (HF) and BPM slope. The diagonal line indicates chance level (AUC = 0.5). The ROC curve is based on out-of-fold predictions obtained through 5-fold stratified cross-validation

**Table 3:** Coefficients of the Final Binomial Logistic Regression Model Predicting Remission Status from ECG-Derived Parameters

Coefficients	Estimate	SE	Z-value	*P*-value
Intercept	1.24003	0.88040	1.408	.1590
BPM slope	−0.48332	0.20150	−2.399	.0165
log(HF)	−0.37695	0.15945	−2.364	.0181

Model fit: residual deviance = 86.554 on 75 df; AIC = 92.554.

**Figure 2:**
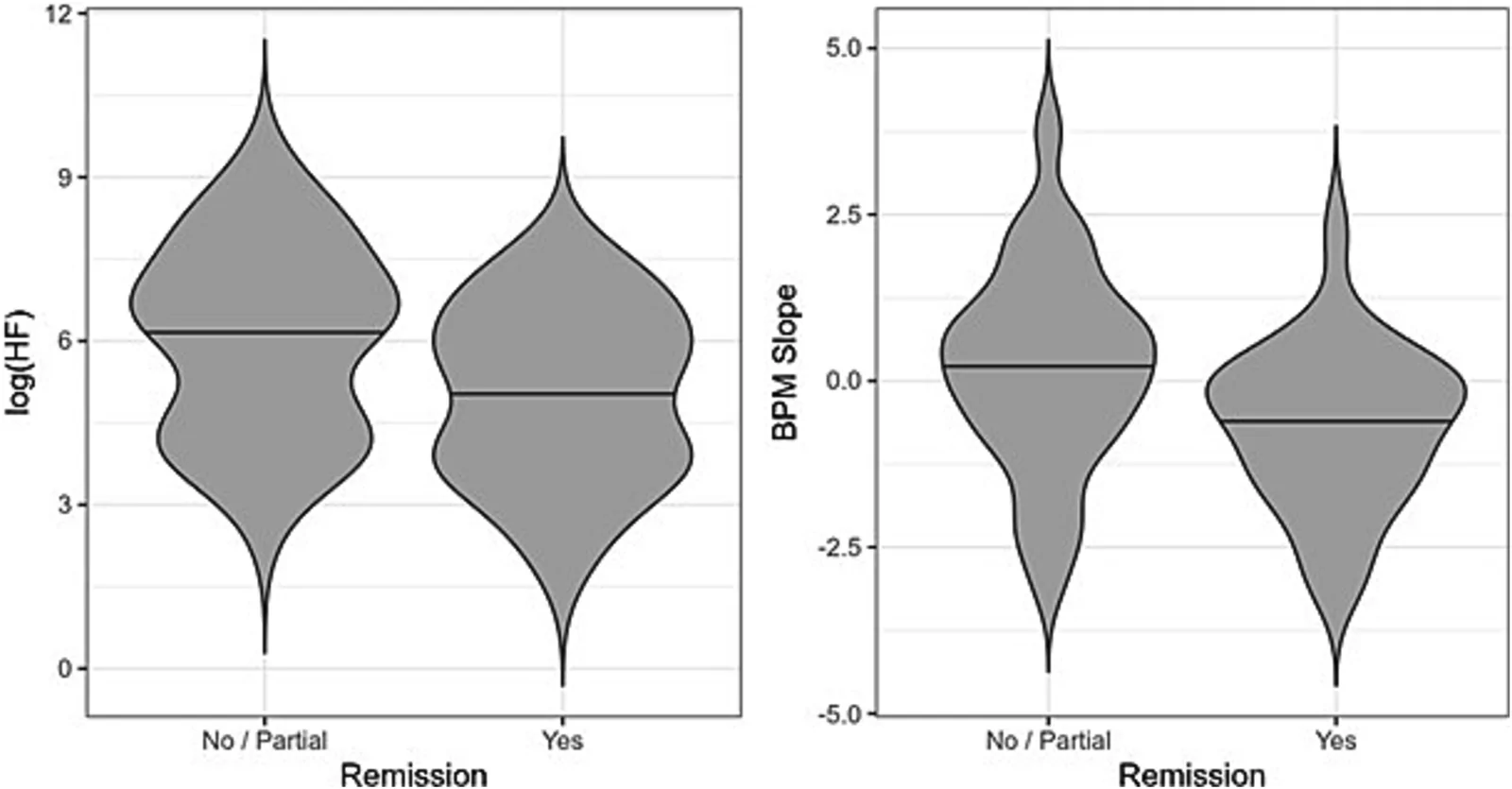
Violin Plots of BPM Slope (Left) and Log-Transformed High-Frequency Power (log(HF); Right) by Remission Status Based Differences in Remission Rates Were Observed (χ^2^ test, *P* > .05)

## Discussion

### Summary of Results and Interpretation

The present study examined whether HRV parameters assessed at hospital admission could predict remission status at discharge in patients with suspected first-episode psychosis. Among the 78 patients included, one-third (33.3%) were classified as being in remission at discharge. No significant sex differences were observed in remission rates or in clinical and physiological measures. In the initial binomial logistic regression model including 4 ECG-derived predictors, log-transformed HF power and the linear change of heart rate over time (BPM slope) were significantly associated with remission status, whereas log(LF) and BPM were not. These findings led to a final model that retained log(HF) and BPM slope as predictors, yielding an AUC of 0.714 based on 5-fold stratified cross-validation. In other words, patients with lower HF power and faster decreasing heart rate at admission had higher odds of remission.

Beyond first-episode psychosis, baseline HRV has also been studied as a predictor of treatment response in other psychiatric disorders. Across depression, anxiety disorders, PTSD, and autism, similar indices (particularly HF power, and in some studies other indices, such as RMSSD and SDNN) have been examined.^23–28^ However, findings are heterogeneous, and recent reviews emphasize that predictive effects vary by diagnosis, treatment modality, subgroup characteristics, and the specific HRV metric.^29^^,^^30^ In psychosis, predictive work remains scarce and differs from the present study in both HRV parameterization and outcomes. Prior studies either related a broad set of classical time- and frequency-domain indices together with nonlinear complexity measures (eg, SDNN, RMSSD, LF, HF, LF/HF, approximate entropy [ApEn], sample entropy [SampEn], corrected shannon entropy) to antipsychotic side effects and treatment adherence,^31^ or examined changes in nonlinear complexity (ApEn/SampEn) in relation to clozapine response in treatment-resistant schizophrenia; notably, baseline differences did not predict response in the latter, whereas week-8 complexity values and their increase over time were associated with better outcomes.^32^ In contrast, our model targets first-episode psychosis and combines a frequency-domain vagal index (HF) with a short-term heart-rate settling measure (BPM slope) at admission to predict symptomatic remission, a dynamic feature not included in previous psychosis studies.

Our findings suggest that specific HRV features—namely, lower HF, which reflects reduced parasympathetic activity and increased physiological tension,^12^^,^^16^^,^^33^ and a more rapidly declining BPM slope, which may reflect greater autonomic relaxation ability—could serve as early indicators of clinical improvement during treatment of first psychotic episodes. While BPM slope is not a standard HRV parameter, a less steep or negative slope over the 2-min ECG may reflect increased capacity for autonomic downregulation in a neutral clinical setting.

HRV is widely regarded as a peripheral window into central autonomic network. From an allostatic perspective,^34^ the cardiovascular system must flexibly allocate metabolic resources to maintain internal stability when confronted with physiological or psychological challenges. Low overall HRV is generally interpreted as a sign of reduced adaptive capacity and heightened allostatic load.^35^^,^^36^ However, the pattern of sympathetic-parasympathetic balance matters: HF reflects respiratory-linked vagal modulation.^37^ Although higher HF is usually considered adaptive, some have argued that excessive vagal tone may become maladaptive under chronic stress^38^ leading to a rigid, energy-conserving state (autonomic fatigue^39^). In that context, our finding that lower HF predicted remission suggests the absence of a parasympathetic dominance at treatment entry, potentially denoting a more adaptive autonomic set-point.^40^ Yet, empirical evidence for such “excessive vagal dominance” is limited and mixed, so this interpretation should be viewed with care. Another explanation could be that patients during an acute psychotic episode often show increased physiological tension, reflected in low parasympathetic and high sympathetic tone. The likelihood of these patients to benefit from first-line treatment, including antipsychotic medication and the safe, structured atmosphere of an inpatient ward, might be higher than in those who already display higher levels of parasympathetic tone and relaxation at admission. This interpretation is supported by previous literature suggesting that schizophrenia is associated with parasympathetic dysfunction, particularly in early psychosis.^41^

On the other hand, the more rapidly declining BPM slope in remitters across the short tracing indicates temporal adaptation: a decrease trend implies rapid settling into baseline once external stressors diminish. Such adaption fits the concept of autonomic flexibility—the capacity to shift effectively between sympathetic mobilization and parasympathetic recovery aligning with the neurovisceral integration model.^33^^,^^42^^,^^43^ This model posits that prefrontal cortical hubs orchestrate rapid, context-appropriate adjustments of autonomic output via cortico-limbic-brainstem loops.^42^ Higher HRV—particularly dynamic shifts, not static levels—indexes the integrity of these loops.

In our data, patients who eventually remitted displayed (i) absence of parasympathetic over-dominance (lower HF) and (ii) fast settling of heart rate, potentially consistent with intact top-down control during a state of increased tension (psychosis). Taken together, the combination of relatively low HF and intact heart-rate recovery may reflect a balanced yet flexible autonomic baseline. This pattern is in line with recent recommendations to consider multiple HRV parameters in combination rather than in isolation. A recent umbrella review emphasized that combining indices such as HF and RMSSD improves clinical relevance and specificity across psychiatric populations.^44^ Although global HRV was lower, patients in remission showed a more balanced sympatho-vagal tone, without global sympathetic nervous system overactivity, which can be interpreted as a physiologically resilient state.^45^

In line with this interpretation, patients who did not go into remission tended to show higher HF and/or less rapidly declining BPM slope patterns. These patterns may indicate a parasympathetic dominance wherein the system might be “locked” in an energy conservation mode,^36^ but lacks the flexibility to re-engage sympathetic drive when new challenges arise. In psychosis, such rigidity could interfere with engagement with psychotherapeutic input, behavioral activation, or medication adherence and drug effectiveness, perpetuating symptom burden.

At first glance, this pattern may appear counterintuitive, since higher HRV is often viewed as a marker of healthier autonomic regulation. However, accumulating evidence suggests that the prognostic role of baseline HRV is not universally linear, but depends on clinical subgroup and treatment context. For example, in randomized trials of CBT for anxiety disorders, lower baseline HRV predicted superior outcomes.^25^ A recent systematic review of anxiety disorders also highlighted mixed findings, with low HRV sometimes linked to better, but in other cases to poorer, treatment response.^29^ Moreover, in generalized anxiety disorder, baseline HF moderated treatment effects: patients with low HF improved more with fluoxetine than with mindfulness-based therapy, whereas this difference disappeared in those with high HF.^26^ Similar observations of low baseline HF predicting better outcomes have been reported in depression (esketamine treatment)^27^ and in autism (response to propranolol).^28^ Together, these findings support that HRV’s prognostic meaning is context-dependent and may, in first-episode psychosis, reflect an acute, plasticity-related imbalance rather than a simple deficit.

In summary, while previous studies have linked reduced HRV to increased symptom severity,^18^^,^^19^ our results point to a potential prognostic role of HRV—beyond symptom correlation—at the onset of treatment. This apparent discrepancy may point to a state–trait distinction: lower HF at admission could reflect an acute, state-related autonomic dysregulation that is amenable to treatment, whereas persistently reduced vagal activity in chronic psychosis might represent a more stable trait marker of unfavorable prognosis. This distinction may reflect different physiological mechanisms underlying symptom burden versus treatment responsiveness, and aligns with the broader view of HRV as a transdiagnostic indicator of regulatory capacity rather than a disorder-specific biomarker,^46^ with potential implications for early clinical decision-making and personalized care.

### Limitations and Strengths

This study has several limitations. First, the findings are preliminary in nature and should be interpreted with caution due to the exploratory design. Given the retrospective, observational nature of the study, all findings are associational; the design does not permit causal inference regarding treatment effects. Second, no detailed information was available regarding the exact medication regimen at the time of ECG assessment at baseline. At this point, autonomic alterations likely reflect both pathophysiology and potential medication effects, although in this first-episode psychosis cohort, substantial antipsychotic exposure was unlikely. Importantly, this combined influence may itself carry prognostic value for subsequent remission. While most evidence points to reduced vagal HRV with antipsychotic treatment, findings have been inconsistent, with some studies reporting no significant medication-related effects. This inconsistency may reflect differences in sample characteristics, antipsychotic agents, dosages, and methodological approaches.^32^^,^^44^^,^^47^ In addition, further factors may influence HRV values and should be considered when interpreting these results. For instance, females typically show lower time-domain HRV and lower LF/HF ratios than males,^14^ although we did not observe sex-related differences in this sample. Additionally, certain cardiac conditions, such as arrhythmias, can artificially inflate HRV values, potentially confounding their interpretation.^48^ Third, treatment followed a naturalistic “therapy-as-usual” approach, based on the clinicians’ discretion, without standardization of pharmacological or psychosocial interventions, and with diagnostic heterogeneity typical of early-episode referrals. Furthermore, widely used research scales such as PANSS, SANS, or SAPS were not systematically collected in this naturalistic setting. Accordingly, remission status was determined based on CGI-S, HoNOS, and clinician ratings, complemented by additional instruments where available. Prior work indicates that CGI ratings correlate strongly with PANSS scores,^49^ supporting the validity of this approach. Moreover, we did not account for other therapeutic or contextual factors that may have influenced outcomes, such as psychosocial interventions, social support, or length of inpatient stay. Fourth, ECG recording covered only a 2-min period, which is less than the suggested 5-min period for short-term recordings.^50^ Lastly, no ECG was performed at discharge, which limited conclusions about potential changes in autonomic function and their relation to clinical improvement. In addition, respiratory parameters were not recorded, limiting the interpretability of frequency-domain measures—particularly high-frequency power, which is strongly influenced by breathing patterns.^51^^,^^52^ In addition, the software used for HRV analysis did not provide classical time-domain indices such as SDNN or RMSSD, which may have added complementary information on autonomic regulation.

This study offers a first step toward evaluating the predictive utility of ECG-derived parameters in first-episode psychosis. The use of non-invasive, cost-effective, and easily applicable physiological markers such as HRV is particularly promising for translation into routine care.

### Clinical and Methodological Implications

Although AUC values between 0.7 and 0.8 are frequently labeled as indicating “moderate” or “acceptable” discrimination, such qualitative classifications vary substantially across studies and lack consistent empirical justification.^53^ Considering this, we report the AUC descriptively without applying a qualitative label. Importantly, even modest predictive accuracy may have clinical value when based on simple, low-cost, and scalable measures.

These exploratory findings provide clinically relevant insights and may inform future approaches to early treatment stratification in first-episode psychosis. Yet, our study did not include additional ECG-derived markers or respiratory recordings, or ECG at discharge, which may have provided complementary information.

### Outlook and Future Directions

Taken together, this study provides preliminary evidence that ECG-based HRV measures may support early identification of patients with favorable treatment trajectories. Namely, lower HF power and a more rapidly declining BPM slope at admission—markers of balanced yet flexible autonomic control—were associated with short-term remission after a first psychotic episode, underscoring the importance of autonomic flexibility rather than absolute vagal tone. Future prospective longitudinal studies with larger samples, repeated HRV and physiological assessments—including respiratory monitoring—and standardized treatment protocols are needed to validate and refine these predictive models and to evaluate treatment effects over time. Additionally, future studies should include both time- and frequency-domain measures to provide a more comprehensive assessment of autonomic function in first-episode psychosis. Ideally, such studies will also examine the integration of HRV features with clinical, behavioral, or digital phenotyping data to improve prediction accuracy and practical utility.

## Data Availability

Data and code can be requested from the corresponding author.
